# Building a Positive Work Environment: The Role of Psychological Empowerment in Engagement and Intention to Leave

**DOI:** 10.3390/bs15020131

**Published:** 2025-01-26

**Authors:** Pachsiry Chompukum, Tita Vanichbuncha

**Affiliations:** 1 Department of Commerce, Chulalongkorn University, Bangkok 10330, Thailand; pachsiry@cbs.chula.ac.th; 2 Department of Statistics, Chulalongkorn University, Bangkok 10330, Thailand

**Keywords:** psychological empowerment, employee engagement, intention to leave, SEM

## Abstract

This study examines the relationships between psychological empowerment, employee engagement, and intention to leave. Employing structural equation modeling, this study analyzes survey data from both academic and non-academic staff in Thailand. Findings reveal that psychological empowerment positively relates to employee engagement, which, in turn, negatively relates to the intention to leave. In addition, psychological empowerment directly affects the intention to leave. This highlights the potential of empowerment-focused interventions as an innovative strategy for improving employee retention, particularly relevant in today’s dynamic and challenging work environments. Our study contributes to the growing body of knowledge on positive organizational psychology by demonstrating the efficacy of empowerment initiatives in a non-Western context. Furthermore, it offers practical implications for organizations seeking nonmonetary rewards to foster a thriving workforce by cultivating a sense of meaning, competence, autonomy, and impact among their employees. Future research can explore the broader applicability of empowerment-based strategies across diverse organizational contexts.

## 1. Introduction

In today’s rapidly evolving business landscape, organizations face unprecedented levels of complexity and volatility, characterized by technological disruptions, geopolitical uncertainties, and shifting market dynamics. This turbulent environment demands continuous innovation and heightened organizational responsiveness to maintain competitive advantage. Within this context, human capital has emerged as a critical determinant of organizational success (Crook et al., 2011), as skilled employees possess the knowledge, capabilities, and adaptability necessary to navigate these challenging conditions. The strategic importance of human capital has consequently elevated talent retention to a paramount concern for organizations worldwide, as the loss of key employees not only disrupts operational continuity but also results in a significant knowledge drain and replacement costs. Organizations must implement effective retention strategies to maintain productivity and sustainability (Patro, 2014). However, recent trends are more challenging with the phenomenon of “quiet quitting”. That is, it is not enough to retain employees, but organizations must go beyond simply retaining employees and focus on keeping them engaged and motivated in order to reduce turnover. Traditionally, organizations have relied on a well-established set of human resource management practices to foster talent retention, including structured career development pathways, formal recognition programs, and competitive compensation packages. While these conventional approaches remain relevant, research suggests that psychological factors play an increasingly pivotal role in employee retention (Avey et al., 2011; Olckers & Du Plessis, 2012, 2015), particularly given the evolving expectations of the new workforce. The modern employee cohort, characterized by different value propositions and career aspirations, places significant emphasis on psychological fulfillment, meaningful work experiences, and emotional connection to their organizations (Martela & Riekki, 2018). This shift in workforce expectations necessitates a more nuanced understanding of the positive work and psychological aspect in organizations. Consequently, psychological empowerment offers a promising approach to addressing the challenges of talent retention and engagement. Additionally, psychological empowerment represents a significant innovation in positive work and organizational psychology by fundamentally shifting from traditional extrinsic control mechanisms to intrinsic motivational states that enhance both employee well-being and organizational effectiveness. It has emerged as a significant factor in enhancing employee retention and engagement. It goes beyond the traditional forms of empowerment, such as giving employees decision-making authority, and focuses on providing them with a sense of control, competence, and meaningfulness in their work. This study aims to investigate the complex interplay between psychological empowerment, employee engagement, and the intention to leave. Specifically, we examine how psychological empowerment shapes employee engagement and subsequently influences turnover intentions, addressing a critical gap in our understanding of employee retention mechanisms. By exploring how psychological empowerment contributes to enhanced employee engagement and reduced turnover intentions, this research offers valuable insights into the psychological dynamics that underpin employee retention. It aims to make a valuable contribution to the literature in the field of employee retention and engagement by emphasizing the critical role of psychological empowerment in creating a positive work environment where employees feel valued, respected, and supported, and where employee morale is high. This will ultimately provide organizations with actionable strategies for creating a supportive work environment that enhances both employee engagement and retention.

## 2. Hypothesis Development

### 2.1. Psychological Empowerment and Employee Engagement

Psychological empowerment refers to the belief and perception that individuals have control over their work and that their efforts make a meaningful impact on organizational outcomes (Dennerlein & Kirkman, 2023; Saira et al., 2021; Spreitzer, 1995). Spreitzer (1995) identifies four key dimensions that constitute psychological empowerment: meaning (alignment between one’s work role and personal beliefs, values, and behaviors), competence (the individual’s belief in their ability to perform work tasks effectively), self-determination (feeling that they have a choice in shaping their work and can exercise a degree of control over their actions and decisions), and impact (the degree to which an employee can influence strategic, administrative, or operating outcomes at work. Through psychological empowerment, employees are able to find significance in their work, feel competent and confident in their abilities, have a sense of autonomy and control over their work, and perceive that their work has a meaningful impact on organizational outcomes. Employees who feel psychologically empowered are more likely to experience a sense of ownership over their work, leading to positive outcomes. Previous research has shown that psychological empowerment is positively related to various work-related outcomes, such as job satisfaction, organizational commitment (Hassard et al., 2022; García-Juan et al., 2018; Seibert et al., 2011), and performance (D’Innocenzo et al., 2015). Psychological empowerment has also been found to be positively related to employees’ creativity. For example, Zhang and Bartol (2010) explored the relationship between empowering leadership and employee creativity, with a focus on the influence of psychological empowerment, intrinsic motivation, and creative process engagement, and they found that psychological empowerment fosters employee creativity. Additionally, Pieterse and colleagues investigated the relationship between transformational and transactional leadership and innovative behavior, highlighting the moderating role of psychological empowerment, and they also found that psychological empowerment is a moderator in the relationship between leadership and innovative behavior (Pieterse et al., 2009). Psychological empowerment is also linked to better psychological well-being and lower job anxiety levels (Marín-García & Bonavía, 2021).

Since psychological empowerment is a collection of four motivational agents—meaning, competency, self-determination, and impact—it is a pivotal factor in enhancing employee motivation and engagement within an organization. Empowered employees, who perceive they have a meaningful impact on their work environment, are often more motivated and driven to positive outcomes. For example, this can be seen in a study which found that empowerment mediated relationships between characteristics and intrinsic motivation among technical and telemarketing workers (Gagné et al., 1997). Employees who perceive they have control over their work and sense that their efforts are impactful exhibit not just compliance, but deep involvement, energy, and enthusiasm toward their job (May et al., 2004; Tsai, 2001). Employee engagement is one manifestation of an employee’s motivation, reflecting how the internal drive to succeed is expressed through their dedication and enthusiasm for their work. Kahn defined employee engagement as “the harnessing of organization members’ selves to their work roles; in engagement, people employ and express themselves physically, cognitively, and emotionally during role performances” (Kahn, 1990). The four elements of psychological empowerment, namely meaning, competence, self- determination, and impact, contribute to the physical, cognitive, and emotional aspects of an employee; they build a sense of purpose, affirm one’s capabilities, provide autonomy, and foster a feeling of making a difference. When employees experience these elements, they are more likely to be actively engaged in their work, displaying enthusiasm and commitment, being proactive, and having pervasive involvement in their tasks and organizational goals. it leads to:

**H1.**  *Psychological empowerment is positively related to employee engagement*.

### 2.2. Psychological Empowerment and Turnover Intention

The intention to leave is a critical issue that spans across various professions. It significantly impacts workforce stability, organizational performance, and the quality of services provided. Employee turnover can disrupt organizational operations, undermine productivity, and compromise the delivery of high-quality services to customers or clients. Factors affecting the intention to leave a job are multifaceted and complex. They can include personal factors and organization-related factors. One personal factor is the individual’s self-concept, which includes task self-confidence and self-esteem. It is found that higher task self-confidence and self-esteem are positively associated with the intention to leave, especially in downsizing organizations (Mone, 1994). The feeling of being personally suited for the job is another personal factor. Employees who perceive a good fit between their personal attributes and job requirements are less likely to leave. Conversely, those who feel mismatched or unprepared for their roles are more likely to consider leaving (Verquer et al., 2003). Although personal factors play a role in turnover intentions, it is the organization-related factors that present more actionable opportunities for retention, as these elements fall within the organization’s sphere of influence. Those factors involve job satisfaction (Hellman, 1997; Tett & Meyer, 1993), organizational commitment (Labatmedienė et al., 2007), opportunities for professional development (Steil et al., 2020), and perceived supervisor support (Cho et al., 2008). One important organizational factor is psychological empowerment. When employees feel psychologically empowered, they experience a sense of ownership and control over their work tasks and outcomes; they also perceive their work as meaningful and valuable and feel competent in their abilities, and believe they can make a significant impact within the organization (Menon, 2001). These empowerment dimensions fulfill employees’ psychological needs, resulting in higher intrinsic motivation. Past research has shown that intrinsic motivation is significantly related to decreased turnover intention. For instance, studies have highlighted the importance of intrinsic motivation in decreasing turnover intention among public sector employees (Kim, 2015, 2018). Furthermore, intrinsic motivation has been found to positively influence individuals’ intention to stay in the education sector (Dysvik & Kuvaas, 2010). Akosile and Ekemen (2022) demonstrated a negative link between staff’s intrinsic motivation and intention to leave. This suggests that individuals who are intrinsically motivated are more likely to remain in their current positions, which is a key driver for wanting to continue in their current role, thus reducing the intention to leave. This leads to the following:

**H2.**  *Psychological empowerment is negatively related to the intention to leave*.

### 2.3. Employee Engagement and Intention to Leave

Employee engagement represents the level of an employee’s emotional and cognitive commitment to their organization, which is characterized by vigor, dedication, and absorption in their work (Schaufeli et al., 2002). Employee engagement plays a vital role in organizational success, as engaged employees not only drive higher productivity and customer satisfaction but also generate additional positive outcomes across various aspects of work performance. For example, engagement is negatively associated with absenteeism; engaged employees are less likely to be absent from work, exhibiting greater vigor and dedication (Neuber et al., 2021). In addition, engaged employees are more likely to exhibit organizational citizenship behaviors, such as helping colleagues and going beyond their job requirements (Akingbola & van den Berg, 2016). Saks (2006) studied the antecedents and consequences of employee engagement and found that it affects organizational commitment and job satisfaction. When employees are engaged, they experience heightened levels of job satisfaction and organizational commitment, developing strong emotional bonds with their workplace through meaningful work experiences and alignment with organizational values. The Affective Events Theory (AET) (Weiss & Cropanzano, 1996) explains how workplace experiences shape employee outcomes through emotional reactions that influence attitudes, behaviors, and decisions. When employees are highly engaged with their organization, they naturally invest more personal resources such as time, energy, and effort into their roles. This investment manifests through an increased initiative in problem-solving and the development of strong professional relationships within the organization. As engaged employees accumulate valuable skills, knowledge, and social connections, they develop significant psychological and social attachments that become barriers to leaving the organization. These barriers arise because the invested resources and established relationships would be costly to abandon and difficult to recreate elsewhere. Given that engaged employees invest considerable personal resources (Xanthopoulou et al., 2009) and develop strong workplace bonds (Walden et al., 2017), they are less likely to consider alternative employment opportunities. Therefore, we hypothesize the following:

**H3.**  *Employee engagement is negatively related to the intention to leave*.

### 2.4. Employee Engagement as the Mediator

While psychological empowerment can directly impact employee turnover intentions, the relationship between psychological empowerment and turnover intentions may not be solely direct. Employee engagement can act as a crucial mediating variable, creating an indirect pathway through which empowerment influences retention. Drawing upon Self-Determination Theory, which emphasizes the human need for autonomy, competence, and relatedness (Deci et al., 2017), psychological empowerment can be seen as a catalyst for fulfilling the fundamental human needs for autonomy, competence, and relatedness within an organizational context. It encompasses various dimensions of an individual’s experience of empowerment (Spreitzer et al., 1997). Unlike general empowerment, which can be broadly defined as the process of gaining power and control over one’s life, psychological empowerment specifically refers to the internal psychological state that reflects an individual’s sense of control, competence, and purpose. Psychological empowerment serves as a mechanism through which employees develop an understanding of the significance and meaningfulness of their responsibilities, a deep belief in their ability to perform competently and achieve successful outcomes, and the perception that they can meaningfully influence the overall organizational outcomes through their efforts. By satisfying these core psychological needs, psychological empowerment fosters a heightened sense of intrinsic motivation (Li et al., 2015) and ownership toward one’s work and directly relates to employee engagement. Engaged employees invest in their roles and experience a sense of purpose. They perceive a positive exchange relationship with the organization, receiving valuable benefits like meaningful work and opportunities for growth. With employees feeling more autonomous and capable, they are likely to become more emotionally and cognitively attached to their job and less likely to look for alternative employment opportunities. While psychological empowerment can directly influence the intention to leave, employee engagement also plays an important role, mediating the impact of psychological empowerment on the intention to leave. This leads to the following:

**H4.**  *Employee engagement mediates the relationship between psychological empowerment and the intention to leave.*

**Figure 1:**
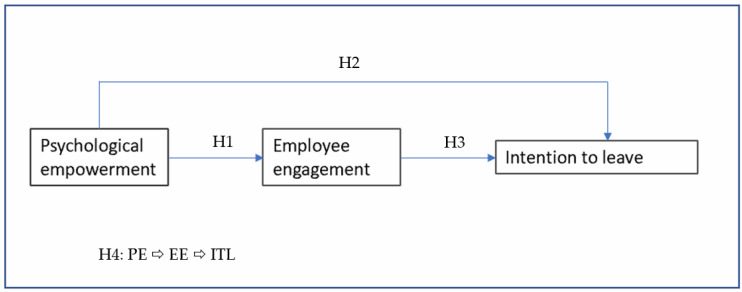
Research model. Source: Prepared by the authors of this study.

In summary, this research develops a model (refer to Figure 1) that examines how psychological empowerment affects employee engagement and the intention to leave.

## 3. Methods

### 3.1. Research Design

This research adopts a cross-sectional research design and employs quantitative analysis to explore the impact of psychological empowerment on employee engagement and the intention to leave. It considers both the direct effect of psychological empowerment on the intention to leave and the mediating effects of employee engagement in this relationship.

### 3.2. Questionnaire Development

Questionnaires used in this study were in two languages, Thai and English. All items are presented in both Thai and English because data were collected in a university that employed Thai and international staff. The original version of all measures is in English. They were translated into Thai using a translation-back-translation procedure (Brislin, 1970). A five-point Likert scale was used to measure the variables, with 1 being strongly disagree and 5 being strongly agree.

### 3.3. Sampling and Measures

To measure employee engagement, forty items were adapted from Kumar and Padhi (2021). This included 9 factors, namely, organization alignment, supervisor’s support and recognition, growth and development, autonomy and opinion, creative and challenging job, job significance, performance evaluation and recognition, welfare, and colleagues and teamwork. An example of items include “my supervisor appreciates my good performance”, “I am always assigned to do tasks that give me chances to acquire new knowledge and skills”, and “My job provides me opportunities to do challenging and interesting work”.

To measure psychological empowerment (PE), the study used 12 items from Spreitzer’s Psychological Empowerment Scale (Spreitzer, 1995), which consists of 4 factors: meaning, competence, impact, and self-determination. Sample items include “The work I do is meaningful to me”, “I am confident about my ability to do my job”, “I have significant autonomy in determining how I do my job”, and “I have a great deal of control over what happens in my department”.

The intention to leave was measured with the three-item scale used by Colarelli (1984). Examples of items are “I frequently think of quitting my job”, and “I am planning to search for a new job during the next 12 months”.

### 3.4. Data Analysis Method

The descriptive statistics and reliability analysis were computed by using SPSS (29th version). Structural equation modeling (SEM) was applied using AMOS (29th version) for data analysis. The SEM is a multivariate analysis tool that assesses the study of Confirmatory Factor Analysis (CFA) and path models with latent constructs simultaneously (Hair et al., 2018). This analysis consists of two steps. Firstly, the first-order CFA was performed to test the validity of the study constructs by using both the Goodness-Of-Fit Index and testing the factor loadings. Moreover, the second-order CFA was conducted to develop the final research structural model. Secondly, the structural model, and the study hypotheses of models were tested with 5% significance level. The R-squared is commonly used to measure performance. An R-squared value of 0.75 is substantial, while 0.5 refers to moderate, and 0.25 refers to weak impact (Hair et al., 2018). However, the Chi-square/DF was not considered here due to the issue of large sample size (Bentler & Bonett, 1980). The alternative fit indices which are less affected by sample size were suggested instead (Kline, 2016).

To test the indirect effect, a bias-corrected bootstrapping procedure with 5000 resamples was employed. The bootstrapping procedure was used to evaluate the significance of the indirect effect (Preacher & Hayes, 2008).

## 4. Results

### 4.1. Common Method Bias

Harman’s one-factor test was conducted to assess the presence of Common Method Bias (CMB) in the study. The results revealed that a single factor accounted for 38.87% of the total variance, which is below the threshold of 50% (Baumgartner et al., 2021), commonly considered indicative of significant CMB. This suggests that CMB is not a concern in this study, and the findings are likely free from any substantial bias related to the method of data collection.

### 4.2. Reliability Analysis

All nine factors from employee engagement, four factors from psychological empowerment, and the single factor from intention to leave demonstrated reliability, as indicated by Cronbach’s alpha values greater than 0.7, suggesting acceptable internal consistency (George & Mallery, 2023), as presented in Table 1.

### 4.3. Descriptive Statistical Analysis

The demographic characteristics of the respondents are summarized in Table 2. The sample was mainly female, with 64.48% of respondents identifying as female and 35.52% as male. In terms of age distribution, the largest age group was between 41 and 50 years, comprising 33.43% of the sample, followed by the 31–40 age group at 29.07%. Participants aged 51–60 years represented 24.97%, while those under 30 years made up 11.06% of the sample, and only 1.48% of respondents were aged over 61 years. Regarding years of service, most participants were employed for 6–10 years (15.73%), 11–15 years (15.85%), or 3–5 years (15.23%). Smaller percentages of respondents reported having worked for less than 1 year (5.40%) or more than 31 years (6.34%). Finally, professional track distribution showed that 64.00% of the respondents were in the Operations Track, while 36.00% were in the Academic Track.

### 4.4. Confirmatory Factor Analysis

To establish whether the measurement model fits the data, both first-order and second-order confirmatory factor analyses (CFA) were conducted using AMOS. Prior to CFA, the dataset’s suitability for factor analysis was assessed. The Kaiser–Meyer–Olkin (KMO) value was 0.97, indicating strong correlations among variables and confirming the appropriateness of the dataset for factor analysis. Additionally, Bartlett’s Test of Sphericity was significant (*p*-value < 0.001), further supporting the suitability of the dataset. The results of the first-order CFA indicated that the measurement model of employee engagement (EE) fit the data well, with all 40 items for measuring employee engagement loading significantly onto their respective factors, which included organization alignment (OA), supervisor’s support and recognition (SSR), growth and development (GD), autonomy and opinion (AO), creative and challenging job (CCJ), job significance (JS), performance evaluation and recognition (PER), welfare (Wel), and colleague and teamwork (CT). The second-order CFA was conducted, and fit indices fell within a good fit, with an Incremental Fit Index (IFI) of 0.94, a Comparative Fit Index (CFI) of 0.94, a Tucker–Lewis Index (TLI) of 0.93, a Goodness-of-Fit Index (GFI) of 0.90, a Root Mean Square Error of Approximation (RMSEA) of 0.05, and a Standardized Root Mean Square Residual (SRMR) of 0.05, for the second-order CFA model of Employee Engagement (EE).

The dataset’s suitability for factor analysis of Psychological Empowerment (PE) was also evaluated. The Kaiser–Meyer–Olkin (KMO) for PE was 0.90, indicating strong correlations among variables and confirming the dataset’s appropriateness for factor analysis. Additionally, Bartlett’s Test of Sphericity was significant (*p*-value < 0.001). The results of the first-order CFA indicated that the measurement model of psychological empowerment (PE) demonstrated a good fit with the data, with all 12 item loadings being significant. The second-order CFA of psychological empowerment (PE) also indicated that the measurement model for Psychological Empowerment (PE) fit the data well, with an Incremental Fit Index (IFI) of 0.97, Comparative Fit Index (CFI) of 0.97, Tucker–Lewis Index (TLI) of 0.95, Goodness-of-Fit Index (GFI) of 0.95, Root Mean Square Error of Approximation (RMSEA) of 0.07, and Standardized Root Mean Square Residual (SRMR) of 0.04.

The standardized factor loadings of the second-order CFA of employee engagement (EE) and psychological empowerment (PE) were evaluated. Most of the standardized loadings were greater than 0.7, indicating strong relationships between the variables and their respective latent constructs (Hair et al., 2018). Some loadings were between 0.5 and 0.7, which are considered acceptable for construct validity (Hair et al., 2018), as shown in Table 3.

### 4.5. Correlation Analysis

The analysis in Table 4 shows that psychological empowerment (PE) is positively correlated with employee engagement (EE) (r = 0.789, *p*-value < 0.001), indicating a strong positive relationship (Cohen, 1988). In contrast, both psychological empowerment (PE) and employee engagement (EE) are negatively correlated with the intention to leave (ITL), with PE showing a moderate negative correlation (r = −0.630, *p*-value < 0.001) and EE demonstrating a stronger negative correlation (r = −0.671, *p*-value < 0.001). These findings suggest that higher levels of psychological empowerment and employee engagement are associated with lower levels of intention to leave.

### 4.6. Hypothesis Testing

To test the proposed hypotheses, a structural equation modeling (SEM) analysis was conducted. The model demonstrated a good fit, with the following fit indices: Incremental Fit Index (IFI) = 0.92, Comparative Fit Index (CFI) = 0.92, Tucker–Lewis Index (TLI) = 0.91, Goodness-of-Fit Index (GFI) = 0.86, Root Mean Square Error of Approximation (RMSEA) = 0.05, and Standardized Root Mean Square Residual (SRMR) = 0.06. These results indicate an acceptable to good fit. The findings, summarized in Table 5, reveal that psychological empowerment (PE) has a positive and significant impact on employee engagement (EE) (b = 0.672, *p*-value < 0.001), supporting hypothesis H1. Furthermore, psychological empowerment (PE) explains a moderate variation in employee engagement (EE), with R-squared = 0.622, indicating that 62.2% of the variation in employee engagement (EE) is accounted for by psychological empowerment (PE).

**Figure 2:**
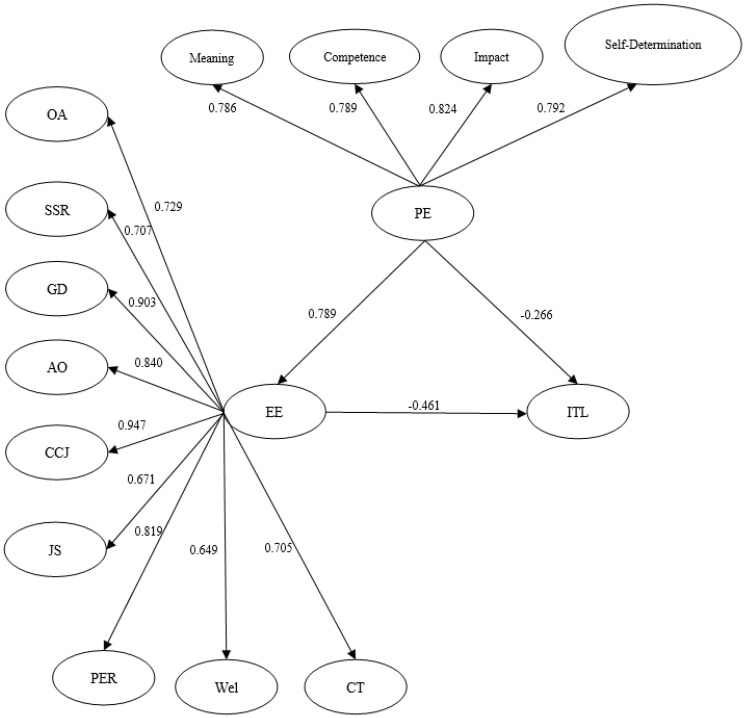
Structural equation modeling (SEM) of intention to leave (ITL), employee engagement (EE), and psychological empowerment (PE). Note: Figure 2 shows standardized values for all path coefficients and factor loadings.

The results for hypothesis H2 indicate that psychological empowerment (PE) has a significant negative impact on the intention to leave (ITL) (b = −0.378, *p*-value < 0.001). This finding supports H2. Employee engagement (EE) was also found to have a negative and significant effect on the intention to leave (ITL) (b = −0.768, *p*-value < 0.001), supporting H3. This finding suggests that higher engagement reduces employees’ likelihood of leaving their jobs. However, the explanatory power of employee engagement (EE) and psychological empowerment (PE) on the intention to leave (ITL) is weak, with R-squared = 0.477, indicating that these variables account for 47.7% of the variation in the intention to leave (ITL). The relationships between psychological empowerment (PE), employee engagement (EE), and intention to leave (ITL), along with their standardized values, are as presented in Figure 2.

To evaluate the mediating effect of employee engagement (EE), the associated *p*-values were calculated using the bootstrapping procedure. The results, presented in Table 6, indicate a significant indirect effect of psychological empowerment (PE) on the intention to leave (ITL) through employee engagement (EE) (*p*-value < 0.001), providing strong support for H4.

Furthermore, the direct effect of psychological empowerment (PE) on the intention to leave (ITL) remained significant in the presence of the mediator, employee engagement (EE). This result indicates partial mediation, meaning that while employee engagement (EE) plays an important role in mediating the relationship between psychological empowerment and the intention to leave, a direct effect of psychological empowerment on the intention to leave persists. Psychological empowerment (PE) influences the intention to leave (ITL) both directly and indirectly through employee engagement. These findings demonstrate that employee engagement (EE) partially mediates the relationship between psychological empowerment (PE) and the intention to leave (ITL). The total effect size of psychological empowerment (PE) and employee engagement (EE) on the intention to leave (ITL) was calculated to be −0.894, indicating a negative impact.

## 5. Discussion

This study investigated the relationship between psychological empowerment, employee engagement, and intention to leave among university staff. The findings offer meaningful insights into the dynamics of psychological empowerment, employee engagement, and their relationship with the intention to leave. Our results revealed a significant positive relationship between psychological empowerment and employee engagement, supporting H1. This suggests that when employees feel a sense of meaning, competence, self-determination, and impact in their work, they are more likely to become engaged. It aligns with prior research that posits empowered employees as more invested, proactive, and committed to their jobs (Sandhya & Sulphey, 2020; Spector, 1986). Our findings also supported H2, indicating a direct negative relationship between psychological empowerment and the intention to leave. This suggests that empowered employees are more likely to stay with organizations, likely due to the increased sense of value and control they experience in their work.

Furthermore, we found support for H3, demonstrating a significant negative relationship between employee engagement and the intention to leave. Engaged employees who are invested in their roles are less likely to consider leaving. This fills a crucial gap in the turnover literature by reaffirming the vital role that engagement plays in retaining talents and underscores the importance of cultivating a work environment that promotes engagement as a retention strategy.

Finally, the results supported H4, revealing a significant indirect effect of psychological empowerment on the intention to leave through employee engagement. This mediating effect suggests that empowerment not only directly influences retention but also indirectly reduces turnover intentions by fostering engagement. This highlights the importance of considering the interplay between these variables when developing retention strategies. Additionally, it extends the existing literature by providing empirical evidence from Thailand’s educational sector, thereby diversifying the predominantly Western-centric knowledge base in this field.

### 5.1. Theoretical Implications

This study advances our understanding of psychological processes in organizations through multiple theoretical lenses by examining the effects of psychological empowerment on employee engagement and the intention to leave.

First, by empirically validating the link between psychological empowerment and employee engagement, this study extends Self-Determination Theory (Deci et al., 2017). Our findings substantiate SDT’s core proposition that fulfilling basic psychological needs for autonomy (through self-determination), competence, and relatedness (through meaning and impact) significantly influences employee engagement and, consequently, organizational outcomes. Specifically, the findings highlight that meaning, by connecting employees to a larger purpose, enhances their sense of belonging and value within the organization. Competence, derived from a sense of mastery and skill development, fosters confidence and intrinsic motivation. Self-determination, by providing autonomy and control over work, empowers employees to take ownership and initiative. Finally, impact, through the perception that one’s work makes a difference, reinforces a sense of purpose and contribution. This nuanced examination of each dimension strengthens SDT’s explanatory power in understanding workplace motivation beyond conventional external reward systems.

Second, the mediating role of engagement reinforces Social Exchange Theory. By enhancing the perceived positive exchange relationship between employees and organizations, psychological empowerment fosters employee engagement and reduces turnover intentions. This exchange is facilitated by each dimension of psychological empowerment: the meaningfulness of work increases the perceived value of the exchange, competence enhances the employee’s contribution to the exchange, self-determination strengthens the sense of reciprocity, and impact reinforces the value of the employee’s contribution to the organization. Engaged employees, feeling valued and empowered, reciprocate by remaining with the organization.

Finally, this study also contributes to the Affective Events Theory (Weiss & Beal, 2005) by demonstrating how positive work experiences, such as feeling psychologically empowered, can lead to positive emotional states like employee engagement, which in turn influence behavioral intentions such as a reduced intention to leave. Specifically, by examining how psychological empowerment, a cognitive motivational state, can evoke positive affective responses like employee engagement, this research extends Affective Events Theory’s explanatory power beyond just discrete emotional reactions to specific work events. The findings suggest that more sustained and holistic positive work experiences, such as feeling a sense of meaning, competence, self-determination, and impact, can shape employees’ overall engagement and their turnover intentions. This nuanced understanding of the effective and behavioral consequences of psychological empowerment contributes to a richer theoretical perspective on the links between work experiences, emotions, and work-related behaviors.

### 5.2. Practical Implications

This study yields significant practical implications for organizations, particularly during periods of economic uncertainty when employee retention and engagement become increasingly critical. The findings underscore the vital role of psychological empowerment in fostering employee engagement within organizational settings. To harness psychological empowerment, leaders should clearly communicate how staff contributions make a difference to organizations and support staff autonomy and development. In addition, management should prioritize creating work environments that enhance employees’ sense of meaning, competence, autonomy, and impact in their roles. This can be achieved through implementing structured professional development programs, designing jobs that align with employees’ values and provide a sense of purpose, involving staff in decision-making processes, and providing clear pathways for career advancement. Human resource management, in particular, plays a pivotal role in this process by integrating psychological empowerment into core HR functions, including recruitment, onboarding, performance management, and training. During recruitment, highlighting opportunities for meaningful work, skill development, and autonomy can attract talent who value empowerment. Onboarding programs should emphasize the impact employees can make within the organization and provide clear expectations for growth and development. Performance management systems should recognize and reward managers who effectively delegate, provide autonomy, and foster a sense of empowerment within their teams. This reinforces the importance of empowerment at all levels of the organization and encourages a top-down approach to fostering a culture of empowerment. Finally, training and development initiatives should focus on building employees’ skills and knowledge, fostering a sense of self-efficacy, and providing opportunities for employees to take ownership of their work. This study suggests that interventions aimed at increasing psychological empowerment can create a positive cascade effect on engagement and retention outcomes. Therefore, organizations should adopt a holistic approach to employee development, integrating empowerment-focused initiatives with engagement-building strategies. Regular assessments of empowerment and engagement levels can help organizations monitor the effectiveness of these interventions and make necessary adjustments. By implementing these practices, organizations can create more sustainable and productive work environments that benefit both the organizations and their employees.

### 5.3. Limitations and Future Research Directions

This study makes a significant contribution to the existing literature on psychological empowerment, engagement, and turnover intentions. However, it is important to note certain limitations that suggest avenues for future research. It was conducted within a single university setting, encompassing both academic and non-academic staff, which provided a comprehensive view of diverse workforce segments within higher education. While this approach offered rich insights into workplace dynamics across different job functions, the institutional context presents natural boundaries for generalization. Furthermore, data came only from employee self-reporting. Future research could incorporate a broader range of data sources such as manager evaluations and organizational records. Future research could investigate additional variables or moderating factors that could influence the central relationships of the study, such as job demand levels, personality traits, or economic cycles.

## 6. Conclusions

This study examined relationships between psychological empowerment, employee engagement, and the intention to leave. Results show a practical psychological approach to employee retention by highlighting how psychological empowerment influences engagement and reduces turnover intentions. It suggests that when employees experience psychological empowerment through meaningful work, competence, self-determination, and impact, they demonstrate higher levels of engagement, which subsequently decreases their intention to leave. This understanding is particularly valuable during periods of economic uncertainty, as organizations seek effective approaches to maintain a committed workforce. While acknowledging that data collection was confined to a single university setting, which may limit generalizability to other organizational contexts, the study’s comprehensive examination of diverse workforce segments provides meaningful insights into workplace psychological dynamics. The study’s implications contribute to theoretical understanding while offering practical approaches for leaders and human resource practitioners in fostering an empowering work environment. Further research could also investigate the influence of moderating factors, such as job demands, personality traits, or economic conditions, on these relationships and incorporate multiple data sources. Overall, this research advances our understanding of psychological empowerment as a key driver of engagement and retention, providing a foundation for developing more effective workplace psychological interventions to promote employee well-being and organizational effectiveness.

## Figures and Tables

**Table 1:** Reliability Analysis.

	Factor	No. of Items	Cronbach’s Alpha
Psychological Empowerment (PE)	Meaning	3	0.914
	Competence	3	0.800
	Impact	3	0.783
	Self-Determination	3	0.879
Employee Engagement (EE)	OA	5	0.862
	SSR	4	0.935
	GD	4	0.863
	AO	4	0.883
	CCJ	4	0.864
	JS	3	0.844
	PER	7	0.910
	Wel	5	0.912
	CT	4	0.896
Intention to Leave (ITL)	ITL	3	0.779

**Table 2:** Demographic characteristics of respondents.

Characteristics	*n*	%
Gender		
Male	1777	35.52
Female	3226	64.48
Age (years old)		
<30	553	11.06
31–40	1454	29.07
41–50	1672	33.43
51–60	1249	24.97
>61	74	1.48
Years of Service		
<1	270	5.40
1–2	340	6.80
3–5	761	15.23
6–10	786	15.73
11–15	792	15.85
16–20	600	12.00
21–25	561	11.22
26–30	571	11.42
>31	317	6.34
Track		
Academic Track	1801	36.00
Operations Track	3202	64.00

Note. Valid %: Based only on cases who answered a question.

**Table 3:** Standardized factor loadings of the second-order CFA.

Contrust	Variable	Standardized Factor Loading
Psychological Empowerment (PE)	Meaning	0.791
	Competence	0.880
	Impact	0.773
	Self-Determination	0.771
Employee Engagement (EE)	OA	0.715
	SSR	0.726
	GD	0.915
	AO	0.832
	CCJ	0.947
	JS	0.641
	PER	0.831
	Wel	0.640
	CT	0.699

**Table 4:** Correlation analysis.

	PE	EE	ITS
Psychological Empowerment (PE)			
Employee Engagement (EE)	0.789		
Intention to Leave (ITL)	−0.630	−0.671	

**Table 5:** Hypotheses testing.

Hypotheses		Estimate	SE	CR	*p*-Value	R-Squared	Conclusion
H1	PE ⇨ EE	0.672	0.02	32.853	<0.001	0.622	Significant
H2	PE ⇨ ITL	−0.378	0.038	−10.071	<0.001	0.477	Significant
H3	EE ⇨ ITL	−0.768	0.048	−15.877	<0.001		Significant

**Table 6:** Mediation analysis.

Hypotheses		Direct Effect	Indirect Effect	*p*-Value	Conclusion
H4	PE ⇨ EE ⇨ ITL	−0.378 (*p*-value < 0.001)	−0.516	<0.001	Partial Mediation

## Data Availability

The data supporting the findings of this study can be obtained by contacting the first author.
